# 

**Published:** 1835-04-01

**Authors:** 


### CONTENTS

OF THE

### MEDICAL QUARTERLY REVIEW,

No. VII. ARPIL 1, 1835.

---

I.

A Treatise on Insanity, and other Disorders Affecting the Mind. By James Cowles Prichard, M.D., F.R.S., &c. . . 1

II.

A Treatise on the Diseases of the Chest, and on Mediate Auscultation. By R. T. H. Laennec, M.D. Translated from the latest French Edition, with copious Notes, by John Forbes, M.D., F.R.S. Fourth Edition; with Plates . . . . . . 33

III.

The Nervous System, Anatomical and Physiological: in which the Functions of the various Parts of the Brain are for the first Time assigned; and to which is prefixed, some Account of the Author's earliest Discoveries, of which the more recent Doctrine of Bell, Magendie, &c. is shown to be at once a Plagiarism, an Inversion, and a Blunder, associated with Useless Experiments, which they have neither understood nor explained. Being the First Volume of an original System of Physiology, adapted to the advanced State of Anatomy. By Alexander Walker, Author of "Physiognomy founded on Physiology" . 46

IV.

Dr. Clark on Tubercular Phthisis: (from the Cyclopœdia of Practical Medicine) . . . . . 50

V.

An Exposition of the Nature, Treatment, and Prevention of Continued Fever. By Henry M'Cormac, M.D. . . . 60

VI.

Observations on the Causes and Treatment of Ulcerous Diseases of the Leg. By J. C. Spender, M.R.C.S. . . . 73

*Reviews continued.*

VII.

Traité complet de l'Art des Accouchemens, &c.

A Complete Treatise on the Art of Midwifery; or, Theoretical and Practical Tocology, with a Compendium of those Maladies which complicate Pregnancy, Labour, and the Puerperal Condition, and of those to which Newborn Infants are liable. Sixteen Plates. By ALFRED VELPEAU, Professor of Clinical Surgery to the Faculty of Medicine at Paris; Surgeon to the Hôpital de la Pitié, &c. Second Edition, corrected and augmented . . . . . . 85

VIII.

Aufsätze und Abhandlungen aus dem Gebiete der Medicin, Chirurgie, und Staatsarzneikunde

Essays and Dissertations on Points of Medicine, Surgery, and State Medicine. By Dr. RUST. Volume the First. With three Lithographic Plates . . . . . . 116

IX.

Outlines of Botany; including a General History of the Vegetable Kingdom, in which Plants are arranged according to the System of Natural Affinities. By GILBERT T. BURNETT, F.L.S., Professor of Botany in King's College, London . . . . 133

X.

Substance of a Clinical Lecture on a Case of Hydrophobia, delivered at the Charing Cross Hospital, Monday, November 24, 1834; to which are appended the Particulars of another Case admitted into the Hospital, October 21, 1834. By T. J. PETTIGREW, F.R.S. &c. Surgeon to the Hospital . . . . . 143

XI.

The Epidemics of the Middle Ages. From the German of I. F. C. HECKER, M.D., Professor at Frederick William's University at Berlin. Translated by B. G. BABINGTON, M.D., F.R.S. No. II. The Dancing Mania . . . . . . 146

XII.

A Treatise on the Formation, Constituents, and Extraction of the Urinary Calculus; being the Essay for which the Jacksonian Prize, for the Year 1833, was awarded by the Royal College of Surgeons in London. By JOHN GREEN CROSSE, Surgeon to the Norfolk and Norwich Hospital 156

XIII.

The Philosophy of Health; or, an Exposition of the Physical and Mental Constitution of Man, with a View to the Promotion of Human Longevity and Happiness. By SOUTHWOOD SMITH, M.D., Physician to the London Fever Hospital, &c. Vol. I. . . . 174

XIV.

An Inquiry into the Nature and Causes of Lateral Deformity of the Spine; in reference, more especially, to the Pernicious Effects of certain Moral and Physical Influences, resulting from the Modern System of Female Education. By EDWARD W. DUFFIN, M.D. Second Edition 178

*Reviews continued.*

XV.

Anatomical Description of the Parts concerned in Inguinal and Femoral Hernia. Translated from the French of M. JULES CLOQUET; with Lithographic Plates from the original Etchings, and a few additional Explanatory Notes. By ANDREW MELVILLE M'WHINNIE, Assistant Teacher of Practical Anatomy at St. Bartholomew's Hospital . 180

XVI.

The Principles of Physiology applied to the Preservation of Health, and to the Improvement of Physical and Mental Education. By ANDREW COMBE, M.D. . . . . . 181

XVII.

Transactions of the Society for the Encouragement of Arts, Manufactures and Commerce, for the Session 1833-1834; being Part I. of Volume L. 183

---

### Original Communications.

I.

On the Causes of some of the Symptoms which attend Diseases and Injuries of the Brain. By Mr. MAYO . . . 185

II.

Some Account of a Tumour, induced by Hæmorrhage, from Rupture of the Nutritious Artery, within the Femur; with Comments on the Treatment. By JOHN HOWSHIP, Esq. (With an Engraving.) . 198

III.

On the Thymus Gland. By W. F. BOW, M.D. . . 207

IV.

MR. KEY's Case of Ulceration of the Cartilages of the Larynx . 214

MR. KEY's Cases of Femoral Hernia and Impenetrable Stricture 216

V.

Structure of the Placenta. Examination of the Hunterian Preparations at the College of Surgeons. From the Medical Gazette; with additional Remarks, by Mr. MAYO. (With an Engraving.) . . 218

VI.

Two Cases of Bronchocele, by JAMES DAMERAM, Esq. . . 223

### COLLECTANEA.

### PATHOLOGY AND PRACTICE.

Dr. Gölis's Treatment of the Diseases of Children . . 225
Dr. Magistel's Cases of Ulceration of the Neck of the Uterus . 240
Dr. Chapman on Tic Douloureux . . . . 243
Case of Atresia of the Vagina, produced by Improper Treatment. By Thomas Jefferson White, m.d. . . . 245
Use of Purgatives . . . . . 246
Cases of Diabetes and Calculus Renalis . . . 247
Diagnosis of Ovarian Dropsy from Ascites . . . 249
Dr. Trousseau on Tracheotomy . . . . 250
Dr. De Leon's Case of Ischuria Renalis . . . 251
Bellingeri on Neuralgia . . . . ib.
Dr. Smith on Tumours of the Neck . . . . 254
A Case of Congenital Deficiency of both the Upper and Lower Extremities. By J. F. E. Hardy, m.d. . . . . 256
Dr. Domeier's Case of Poisoning by Fish . . . 257
Mr. Chevallier on Syrup of Rhubarb Stalks . . . 258
Dr. Strahl's Experiments on the Effects of Indigo in Spasmodic Diseases 259
M. Guersent on Belladonna in Hooping-cough . . ib.
Dr. Tradini on the Use of Camphor in Tympanitis . . 260
Dr. Strahl on Stammering and its Cure . . . 261

### MISCELLANEOUS.

M. Geiger on the Varieties of Rhubarb . . . 264
Mr. Kyan's Patent for the Preservation of Wood by Corrosive Sublimate 264
M. Archard on the Chemical Effects of Electricity . . ib.
Dr. Renton on the Climate of Madeira . . . ib.
Dr. Cummin on Medico-Legal Disinterments . . . 266
Cases of Persons falsely Imprisoned as Lunatics . . 269

Meteorological Register. . . . . . 498
Notices . . . . . . ib.

